# Serum Levels of Interleukin-8 and Soluble Interleukin-6 Receptor in Patients with Stage-I Multiple Myeloma: A Case-Control Study

**DOI:** 10.31557/APJCP.2020.21.1.127

**Published:** 2020

**Authors:** Maryam Kohsari, Mohammad-Hassan Khadem-Ansari, Yousef Rasmi, Hojjat Sayyadi

**Affiliations:** *Department of Biochemistry, Faculty of Medicine, Urmia University of Medical Sciences, Urmia, Iran. *

**Keywords:** Multiple myeloma, interleukin-8, soluble interleukin-6 receptor

## Abstract

**Objective:**

Multiple myeloma (MM) remains an incurable disease that needs better recognition and further research. Previous studies elucidated the interaction between myeloma cells and showed the necessity of bone marrow stromal cells for the initiation and progression of MM. Many chemokines and their receptors including interleukin-8 (IL-8) and soluble interleukin-6 receptor (sIL-6R) play important roles in this interaction. The main purpose of this study is evaluating the serum level of IL-8 and sIL-6R on stage-I of MM patients and healthy controls.

**Methods:**

Serum samples from 30 stage-I MM patients (13 males and 17 females) and 30 healthy subjects as controls (13 males and 17 females) were examined in this study. The protein concentrations of serum IL-8 and sIL-6R were assessed by enzyme-linked immunosorbent assay (ELISA).

**Results:**

The mean level of IL-8 and sIL-6R were significantly elevated in stage-I MM. The mean levels of IL-8 were 1246.57±279.22 ng/ml in stage-I MM and 902.53± 294.61 ng/ml in controls (P<0.001). The mean levels of sIL-6R were 5.39±1.38 ng/ml and 4.1±1.14 ng/ml in stage-I MM and controls, respectively (P<0.001). The mean levels of IL-8 were 1342.18±193.4 ng/ml in patient females and 859± 278.2ng/ml in control females (P <0.001). The mean levels of sIL-6R were 5.21±1.55 ng/ml and 3.91±1.22 ng/ml in patient females and control females, respectively (P=0.01). The mean level of sIL-6R in patient males and control males were 5.63±1.43 ng/ml and 4.34±1.04 ng/ml, respectively (P=0.01). A significant correlation (Pearson’s correlation = 0.45, P=0.008) was observed in the population of females (patients and controls).

**Conclusion:**

The results of study suggest the possible involvement of IL-8 and the sIL-6R at stage-I MM and can better characterize the role of chemokines and their receptors in the disease process, especially in the early stages.

## Introduction

Multiple myeloma (MM) accounts for almost 1% of all cancers and 10% of hematological malignancy (Alexanian and Dimopoulos, 1994; Kastritis et al., 2009). This disease still remains incurable (Bustoros et al., 2017) and is characterized by increasing monoclonal immunoglobulin secreted by clonal malignant plasma cells within the bone marrow (BM) and the clinical evidence such as osteolytic bone lesions, hypercalcemia, renal disease, and anemia (Coleman, 1997; Mahindra et al., 2010). The interaction between myeloma cells and bone marrow stromal cells (BMSCs) is crucial to homing, proliferation, migration, and drug-resistant (Aggarwal et al., 2006). Clearly, myeloma cells and BMSCs both express a variety of chemokine receptors. Moreover, they produce various chemokines by the interaction of chemokine receptors and their ligands in BM- microenvironment, which promotes the progression of MM (Wallace et al., 2001).

Interleukin-8 (IL-8) belongs to the alpha chemokine family of cytokines (Yoshimura et al., 1987) and has a chemotactic effect on neutrophils, eosinophils, basophils, monocytes, and B lymphocytes (Strieter, 2002). The overexpression in a variety of tumors has led to investigating its role in tumor progression (Green et al., 1997). In this regard, several studies have illustrated the role of IL-8 in angiogenesis, cell motility, and invasion in tumor metastasis (Sozzani et al., 1996; Belperio et al., 2000; Mukaida, 2000). Interleukin-8 is secreted by endothelial cells and BMSCs in MM (Merico et al., 1993; Pellegrino et al., 2005). It is hypothesized that IL-8 attracts malignant plasma cell precursors that are in the blood into an interleukin-6 rich bone marrow microenvironment (Lauta, 2003). In addition, IL-8 stimulates osteoblast proliferation and bone resorption by regulating receptor activator of nuclear factor kappa-B ligand (Bendre et al., 2003).

Interleukin-6 is the main growth and survival factor of myeloma cells and is over-produced in the form of paracrine by BMSCs in contact with myeloma cells (Anderson and Carrasco, 2011). Interleukin-6 induces its effect through its specific receptor at the target cell surface (Taga and Kishimoto, 1997). Soluble interleukin-6 receptor (sIL-6R) is the alternatively spliced cleaved form of interleukin-6 receptor that lacks the transmembrane domain but is able to mediate the effect of IL-6 through the trance-signaling pathway (Rose-John and Heinrich, 1994; Jones et al., 2011). In this context, it has been reported that there is a connection between elevated levels of sIL-6R and poor prognosis and shorter survival in MM patients (Kyrtsonis et al., 1996; Stasi et al., 1998). In this study, we postulated that IL-8 and sIL-6R may be elevated in stage-I of MM patients and contribute to the progression of the disease. To explore this issue, we performed a case-control study to assess the serum level of IL-8 and sIL-6R in stage-I of MM patients and healthy controls. 

## Materials and Methods

*Patient and control*

The study population includes 30 diagnosed stage-I MM patients (13 males and 17 females; with mean age 64.9±7.2 years) and 30 healthy controls (13 males and 17 females; with a mean age of 64.6±6.5 years) used as controls.

*To confirm patients are stage-I MM, several experiments were performed*

Laboratory tests including the complete blood count (CBC) for examining the anemia, erythrocyte sedimentation rate (ESR) that must be in patient more than 80 mm/hour, blood chemistry tests for evaluated serum level of creatinine to check kidney function, albumin, calcium, and lactic dehydrogenase (LDH) (Pars Azmun, Tehran, Iran) were used to measure all factors of blood chemistry. Next, β2-microglobulin (LIAISON®, DiaSorin, Italy) was measured and serum capillary zone electrophoresis (Capillary^®^, Sebia, France) was performed to see the peak in the gamma area. Moreover, immunotyping was carried out to detect the type of light chains (kappa or lambda). The bone marrow tissue biopsy was taken from the patients to examine the size and shape of the cells and see how the cells are arranged. The criteria to diagnose MM is observing at least 10% of plasma cells in the bone marrow biopsy.

In the end, the results of the experiments were evaluated with the International Staging System (ISS)(Greipp et al., 2005) and the stage of the disease was determined. Patients with advanced stages were excluded from the study. Also, none of the patients had received chemotherapy or radiotherapy prior to sampling. Healthy controls were examined for the absence of inflammatory diseases and other underlying conditions that could affect the outcome of the study. Eventually, the samples that confirmed the lack of evidence of impairment entered the study.

*Blood sampling*

Blood samples were obtained through overnight fasting. The serum was separated via centrifuge at 300 g for 10 min. Then, it was transferred to the cryotubes and stored at -70^o^C and assayed at the end of the study, to avoid inter-assay variability. 

*Measurement of IL-8 and sIL-6R*

The serum level of IL-8 was measured by a quantitative sandwich enzyme immunoassay method using Bioassay Technology Laboratory (Shanghai Crystal Day Biotech Co., Ltd). Briefly, a monoclonal antibody specific for IL-8 was pre-coated onto a microplate. Standard and samples were pipetted into the cells. After washing, an enzyme-linked monoclonal antibody specific for IL-8 was added into the wells. Upon adding a substrate solution, the intensity of the color, which develops in each well in proportion to the amount of IL-8 bound during the initial step, is measured by performed ELISA reader at 450 nm and 630 nm wavelength. In the same way and according to manufacturer instruction, serum level of sIL-6R was measured in accordance with the instructions given in (Shanghai Crystal Day Biotech Co., Ltd).

*Statistical methods*

Data analysis was performed by SPSS software (SPSS Inc. Released 2007. SPSS for Windows, Version 16.0. Chicago, USA). All measured parameters are expressed as mean ± SD. Before the analysis, the data were checked for normality with the Kolmogorov-Smirnov test. The parametric Independent-Samples T-test was used to perform statistical comparisons between the stage-I of MM patients and controls. Correlation between the measured parameters was calculated by Pearson’s correlation. P-values <0.05 were considered as statistically significant. 

**Table 1:** Demographic Features and Clinical Characteristics of MM and Controls

	MM	Controls	P-value
Age (year)	64.9±7.2	64.6±6.5	0.83
Gender (male/female)	13/17	13/17	1.00
Hemoglobin (g/dl)	12.62±0.89	13.10±1.2	0.82
ESR (mm/hour)	86.53±5.14	19.73±13.26	< 0.001
Calcium (mg/dl)	9.03±0.88	8.80±0.84	0.30
Creatinine (mg/dl)	0.98±0.18	0.86±0.17	0.01
LDH (u/l)	176.36±31.19	165.83±26.87	0.16
Albumin (g/dl)	3.91±0.52	5.02±0.35	< 0.001
β_2_-microglobulin (mg/dl)	1.72±0.66	1.58±0.49	0.35

**Figure 1:**
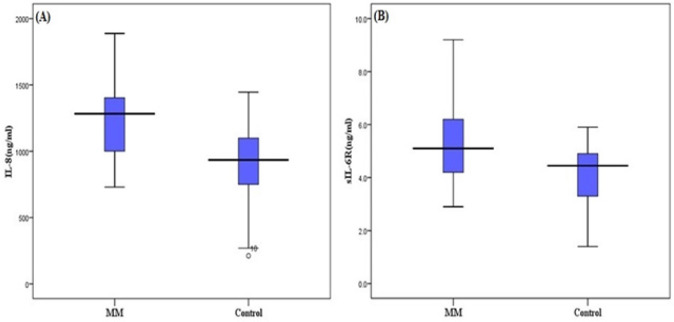
Distribution of Serum Level of IL-8 (A) and sIL-6R (B) between MM (multiple myeloma patients) and Controls. A significant difference is observed (P< 0.001).

**Figure 2:**
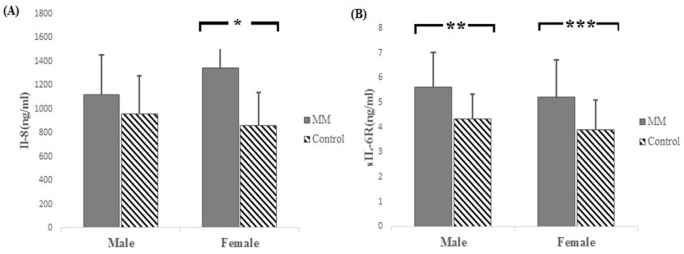
Comparison of the Mean Level of IL-8 (A) and sIL-6R (B) in the Population of Males and Females. Results show a significant difference in the mean level of IL-8 in females (*P< 0.001). The mean level of sIL-6R is significantly different in both males (**P=0.01) and females (***P=0.01).

**Figure 3:**
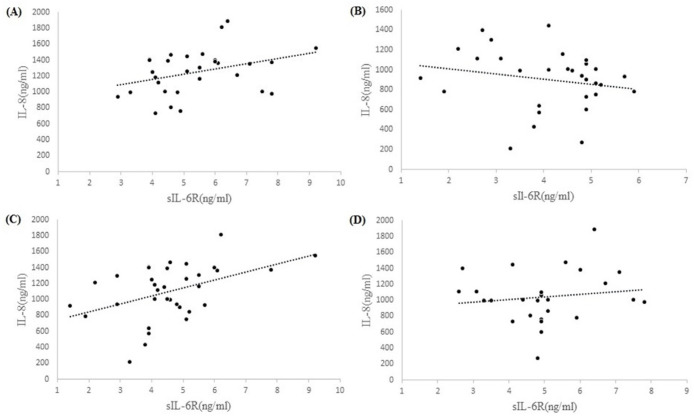
Correlation between IL-8 and sIL-6R in MM (A), Controls (B), Population of Females (C) and Population of Males (D). The results show significant correlation in population of females (Pearson’s correlation =0.45, P=0.008). Correlations are in patients (Pearson’s correlation =0.34, p=0.06), controls (Pearson’s correlation = -0.20, P= 0.28), and population of males (Pearson’s correlation =0.13, P=0.51).

## Results

Demographic features and clinical characteristics of patients and healthy controls are reported in Table 1. The mean serum level of IL-8 in stage-I MM patients was 1246.57±279.22 ng/ml, although it was higher than the mean of controls, which is 902.53± 294.61ng/ml (P<0.001) (Figure 1). A similar result was observed for sIL-6R such that the mean serum levels of sIL-6R in patients and controls were 5.39±1.38 ng/ml and 4.1±1.14 ng/ml, respectively (P<0.001) (Figure 1). 

There was also a significant difference between the mean level of IL-8 and sIL-6R among patient females and control females. The mean IL-8 in patient females was 1342.18±193.4 ng/ml while in the control females, it was 859± 278.2ng/ml (P<0.001). Moreover, the mean sIL-6R in patient females and control females was 5.21±1.55 ng/ml and 3.91±1.22 ng/ml, respectively (P=0.01). In the population of males, a significant difference was observed in the mean serum level of sIL-6R between patient males (5.63±1.43 ng/ml) and control males (4.34±1.04 ng/ml) (P=0.01) (Figure 2). 

The results showed no significant correlation between IL-8 and sIL-6R with any of the measured parameters and also no significant correlation between IL-8 and sIL-6R in patients (Pearson’s correlation =0.34, P=0.06), controls (Pearson’s correlation = -0.20, P= 0.28), and males population (Pearson’s correlation =0.13, P=0.51). Only a significant correlation was observed between IL-8 and sIL-6R in the population of females (Pearson’s correlation =0.45, P=0.008) (Figure 3).

## Discussion

Multiple myeloma is a cancer of plasma cells resulting from the abnormal proliferation of malignant plasma cells within the BM microenvironment (Coleman, 1997). Despite the advancement in the detection and emergence of new treatments, this disease remains still incurable (Bustoros et al., 2017). The exact cause of the disease has not been known until now (Angtuaco et al., 2004). However, a deeper understanding of the molecular mechanisms of MM growth, survival, and resistance to therapy, as well as the interaction between MM cell and BM microenvironment will provide the framework for the development of novel therapies to further improve the outcome of patients. In our previous studies, we focused on the role of stress oxidative in Stage-I MM and show that oxidative stress are considered in the primary pathogenesis of MM diseases (Faridvand et al., 2016; Khadem-Ansari et al., 2017). To the best of authors’ knowledge, there is no study on serum level of IL-8 and sIL-6R, especially on stage-I MM. In this study, our goal was to investigate the changes in serum level of Interleukin-8 and sIL-6R in stage-I MM patients. IL-8 promotes cancer cell growth, survival, angiogenesis, and metastasis in several tumors (Arenberg et al., 1996; Luca et al., 1997; Singh and Varney, 2000; Kim et al., 2001). In MM, endothelial cells and BMSCs produce overexpression of IL-8 (Merico et al., 1993; Pellegrino et al., 2005). According to a hypothesis, IL-8 may play a role to attract circulating malignant plasma cell precursors existing in the blood into an IL-6-rich bone marrow microenvironment (Lauta, 2003). Our results show that increased serum levels of IL8 in patients compared to healthy controls. In one study, Herrero et al., (2016) showed the effects of IL-8 up-regulation on osteoclast genesis in MM. The results are also proven in the breast cancer cell line (Gür et al., 2002; Kristo et al., 2002; Martin, 2002). Other previous studies have revealed the similar role of interleukin-8 in other cancer cell lines. IL8 correlates with angiogenesis in gastric carcinoma (Kitadai et al., 1999) and colon cancer (Fidler, 1997). IL-8 has also been shown to be an autocrine growth factor for melanoma cell and human liver and pancreatic cancer (Miyamoto et al., 1998). The role of IL-8 in metastasis has also been identified in melanoma cancer (Singh et al., 1994), gastric carcinoma (Kitadai et al., 2000), ovarian cancer (Xu and Fidler, 2000), and prostate cancer (Greene et al., 1997). Our data demonstrate an increased level of sIL-6R in patients compared with a healthy control group. Studies on sIL-6R in MM represent the role of this factor in bone resorption by stimulating osteoclast formation (Tamura et al., 1993). Moreover, it was reported that the circulating levels of sIL-6R can act as a useful prognosis marker since high serum sIL-6R concentrations were associated with patients who died within 3 years of diagnosis (Pulkki et al., 1996). Although we found a correlation between IL-8 and sIL6R only among the population of females, these two factors have similar roles in bone destruction as the most important complications and causes of the disease (Mundy et al., 1974; Walker et al., 2007). It has been show that sIL-6R is involved in inducing interleukin-6 activity (Taga and Kishimoto, 1997). In this regard, there is clear evidence to support that both autocrine and paracrine mechanisms of IL-6 and IL-8 production have a correlation with the promotion of MM cell growth, survival, and migration, and angiogenesis in the BM- microenvironment (Zhu et al., 2003; Ndlovu et al., 2009). Considering the existing evidence and in order to clarify the process of MM disease, however, further research is needed on these two factors. 

In conclusion, the results of the study suggest that IL-8 and sIL-6R are involved in the development of MM in stage-I. Regarding the need for better understanding of the disease, especially in the first stage, the results demonstrate the role of chemokines and their receptors in the disease process in stage I of MM. Furthermore, this two factors can be used for choice better therapeutic strategies.
